# Multilevel factors associated with self-reported sexually transmitted infections among sexually active women in Africa: a socio-ecological analysis

**DOI:** 10.1186/s12889-026-28423-9

**Published:** 2026-07-09

**Authors:** Ebenezer Kwesi Armah-Ansah, Eugene Budu, Cyprian Issahaku Dorgbetor, Rabbi Tweneboah, Lucy Faustina Ladzro, Emmy Kageha Igonya, Elom Hillary Otchi, Charity Oga-Omenka

**Affiliations:** 1 School of Public Health Sciences, Faculty of Health, University of Waterloo, Waterloo, Canada; 2 JC School of Public Health and Primary Care, The Chinese University of Hong Kong, Hong Kong, Hong Kong S.A.R., China; 3 Department of Economics, Kwame Nkrumah University of Science and Technology, Kumasi, Ghana; 4 Department of Psychology and Pedagogy, People’s Friendship University, Moscow, Russia; 5 Sexual Reproductive Maternal Neonatal Child and Adolescents Health Unit, African Population and Health Research Center, Nairobi, Kenya; 6 Quality Management Unit, Africa Institute of Healthcare Quality, Safety & Accreditation (AfIHQSA), Accra, Ghana; 7 Community Care Department, Accra College of Medicine, Accra, Ghana

**Keywords:** DHS, Prevalence, Public health, Sexually active women, Africa, STIs

## Abstract

**Background:**

Sexually transmitted infections (STIs) continue to pose a significant public health challenge globally, negatively impacting reproductive health and increasing the likelihood of infertility, complications during pregnancy, and mother-to-child transmission. Despite the increasing burden of self-reported (SR) STIs, there is limited evidence on their prevalence and associated factors across Africa. Applying the socio-ecological model (SEM), this study sought to estimate the prevalence and identify the factors associated with SR-STIs among sexually active women in Africa.

**Methods:**

The study used the most recent Demographic and Health Surveys (DHS) conducted in 29 African countries. A weighted sample of 340,179 sexually active women who had ever had sexual intercourse in the past 12 months and had complete information on all variables of interest were included. Guided by SEM, explanatory variables were conceptually mapped onto its four levels (individual, interpersonal, community, and societal/structural). The analysis was conducted using Stata version 14.2. Descriptive, chi-square, and multilevel regression analyses were carried out. Frequencies, percentages, and odds ratios (ORs) with corresponding 95% confidence intervals (CIs) and *p*-values were used to present the results.

**Results:**

The pooled prevalence of SR-STIs among sexually active women in Africa was 18.43% (95% CI: 18.00%–18.70%), ranging from 47.9% in Liberia to 3.9% in Ethiopia. Guided by the SEM levels, significant associations were identified at the individual, interpersonal, and societal/structural levels. At the individual level, women aged 45–49 [aOR = 0.71; 95% CI = 0.66–0.76] and women whose sexual debut occurred at age 20 or older [aOR = 0.76, 95% CI = 0.73–0.79] had lower odds of SR-STIs than women aged 15–19 years and those whose sexual debut occurred before age 20 years, respectively. At the interpersonal level, women living in female-headed households had lower odds of SR-STIs [aOR = 0.96, 95% CI = 0.93–0.99]. At the societal/structural level, women in Southern Africa had lower odds of SR-STIs [aOR = 0.35; 95% CI = 0.32–0.39] than those in West Africa.

**Conclusion:**

This research revealed a high prevalence (18.43%) of SR-STIs among sexually active women in Africa, with substantial cross-country and sub-regional variation. The findings demonstrate the utility of the SEM in understanding SR-STIs, as significant factors were identified across individual, interpersonal, community, and societal/structural levels, highlighting that SR-STI risk is shaped by a complex interplay of multilevel determinants. These findings call for targeted, context-specific interventions that address factors at multiple levels, including enhancing STI education and screening as part of routine reproductive health and HIV services. Additionally, governments should implement region-specific strategies to improve reproductive health outcomes.

## Introduction

HIV, viral hepatitis, and sexually transmitted infections (STIs) remain major global public health challenges [1]. Despite substantial advances in policy, scientific research, diagnostics, prevention, and treatment, STIs continue to affect millions of people globally [2–5]. Among people aged 15–49 years, more than one million new STIs occur each day, with an estimated 374 million new cases of chlamydia, gonorrhea, syphilis, and trichomoniasis occurring in 2020 alone [6]. This burden is disproportionately higher in low- and middle-income countries (LMICs), particularly in Africa, where the estimated annual number of STI cases is approximately 96 million [1].

In response to this sustained global burden, the World Health Organization (WHO) has set targets to reduce the incidence of STIs by 2030, calling for the scale-up of national prevention and control strategies [7]. These measures, which are in line with the Sustainable Development Goals (SDGs), focus on improving surveillance, prevention, early diagnosis, and targeted actions for at-risk groups, all within a framework that promotes human rights and gender equality [8].

Sexually transmitted infections not only cause immediate health issues but are also linked to adverse social, economic, and health consequences, including pelvic inflammatory disease (PID), infertility, and illnesses, as well as increased susceptibility to HIV infections [9–11]. Women face a higher risk of both acquiring STIs and experiencing their complications due to biological factors, a greater chance of having asymptomatic infections, and social and sexual dynamics that increase their exposure risk [3, 12–15]. These risks are worsened by ongoing diagnostic challenges in many African health systems, such as insufficient and costly diagnostics for asymptomatic infections and drug resistance, misdiagnosis, and low-test sensitivity [6, 16]. Practitioners in Africa and other LMICs rely on syndromic management using clinical algorithms (based on observed symptoms such as vaginal/urethral discharge) for rapid and simple detection and treatment. However, as highlighted in several studies [17–20], syndromic management can sometimes be ineffective in diagnosing STIs, particularly asymptomatic ones. It can often lead to either overtreatment or missed detection of asymptomatic STIs [1, 6]. Untreated STIs among women can lead to serious long-term outcomes, including ectopic pregnancy, chronic pelvic pain, congenital infections, and tubal infertility, which are also strongly associated with poor mental health outcomes, stigmatization, and intimate partner violence [21–26].

In this context, self-reported (SR) STIs represent a pragmatic approach for understanding the STI burden in the Africa region [3, 9]. Self-reporting STIs can support targeted prevention and control strategies and help identify populations at higher risk of STIs where syndromic management alone may be insufficient [26]. Therefore, research on the prevalence and factors associated with SR-STIs does not only add to literature in Africa but can help facilitate targeted interventions and efficient allocation of resources. Therefore, this study assesses the prevalence of SR-STIs and utilizes socio-ecological model to investigate the factors related to SR-STIs among sexually active women.

## Theoretical framework

This research was guided by the socio-ecological model (SEM) to investigate the prevalence and associated factors of SR-STIs among sexually active women in Africa. The SEM provides a comprehensive framework for understanding how multiple interacting factors across different levels influence health outcomes, particularly SR-STIs. The SEM recognizes that individual behaviors are shaped by complex interactions between personal, interpersonal, community, and societal factors [27, 28].

The SEM has been used in various health settings to analyze healthcare usage and health-related behaviors [29–31]. By applying the SEM, this study provides a comprehensive understanding of how multilevel factors collectively influence self-reported STIs among sexually active women across 29 African countries.

## Materials and methods

### Data source and study population

This study used secondary data obtained from the most recent Demographic and Health Surveys (DHS) conducted between 2011 and 2024 in 29 African countries. The datasets were accessed from the DHS Program database (https://dhsprogram.com/data/available-datasets.cfm). The surveys spanned more than a decade, with the earliest dataset being from Congo (2011–2012) and the most recent datasets from Angola, Lesotho, Mali, Nigeria, and Zambia (2023–2024) (Table 1). The countries were grouped into four sub-regions: Southern Africa (Lesotho, Namibia, and Zimbabwe); East Africa (Burundi, Comoros, Ethiopia, Madagascar, Malawi, Mozambique, Rwanda, Uganda, and Zambia); West Africa (Benin, Burkina Faso, Côte d'Ivoire, The Gambia, Ghana, Guinea, Liberia, Mali, Nigeria, Sierra Leone, and Togo); and Central Africa (Angola, Cameroon, Chad, Congo, the Democratic Republic of the Congo, and Gabon).

**Table 1:** Detailed description of the study sample

No	Survey country	Year of survey	Weighted sample (N)	Weighted %
1.	Angola	2023–2024	12,835	3.77
2.	Benin	2017–18	13,975	4.1
3.	Burkina Faso	2021	15,136	4.45
4.	Burundi	2016–2017	12,013	3.5
5.	Cameroon	2018	11,469	3.37
6.	Chad	2014–2015	5,108	1.5
7.	Comoros	2012	3,729	1.1
8.	Congo	2011–12	10,150	2.98
9.	Congo DR	2013–14	16,435	4.8
10.	Cote d’Ivoire	2021	13,214	3.88
11.	Ethiopia	2016	11,962	3.5
12.	Gabon	2012	5,640	1.66
13.	Gambia	2019–20	8,824	2.59
14.	Ghana	2022–2023	12,997	3.8
15.	Guinea	2018	9,162	2.69
16.	Lesotho	2023–2024	2,583	0.76
17.	Liberia	2019–20	7,559	2.2
18.	Madagascar	2021	16,823	4.95
19.	Malawi	2015–2016	21,589	6.35
20.	Mali	2023–2024	14,579	4.29
21.	Mozambique	2022–2023	11,961	3.5
22.	Namibia	2013	7,804	2.29
23.	Nigeria	2024	25,639	7.5
24.	Rwanda	2019–20	10,590	3.1
25.	Sierra Leone	2019	13,965	4.1
26.	Togo	2013–14	8,321	2.45
27.	Uganda	2016	15,809	4.65
28.	Zambia	2024	12,244	3.6
29.	Zimbabwe	2015	8,064	2.37
	**African countries**	**2011–2024**	**340,179**	**100.0**

Countries were included if they met the following criteria: (1) the most recent DHS was conducted between 2011 and 2024; (2) data were available on self-reported STI symptoms (genital discharge, genital sore/ulcer, or reported STI diagnosis); (3) the survey contained complete information on the outcome and explanatory variables; and (4) respondents were sexually active women aged 15–49 years who reported sexual intercourse within the 12 months preceding the survey. Countries whose most recent DHS was conducted before 2011 were excluded to ensure comparability and contemporariness of pooled estimates across countries. Based on these criteria, 29 countries were included in the final analytic sample.

We used data from the women's files (ages 15–49 years). The DHS is a nationally representative cross-sectional survey program that collects data approximately every five years [32, 33]. The final analytic sample comprised 340,179 women (Table 1). We followed the Strengthening the Reporting of Observational Studies in Epidemiology (STROBE) guidelines in drafting this manuscript [34].

### Description of variables

#### Outcome variable

The outcome variable in this study examined whether sexually active women in Africa self-report sexually transmitted infections in the past 12 months prior to the various survey years. This variable was derived from three questions in the DHSs *(had any STI in the last 12 months; had genital discharge in the last 12 months; and had a genital sore or ulcer in the last 12 months)*. The response options were *“no, yes, and don’t know”*. In this study, the responses were categorized into “No” (no, and don’t know) to be “0” and “Yes” (yes) to be “1”.

#### Independent variables

Seventeen independent variables were selected for this study based on empirical evidence from previous studies and the guiding principles of the socio-ecological model (SEM) [35–38]. The SEM posits that health outcomes are shaped by interactions between factors operating at multiple levels, including individual, interpersonal, community, and societal/structural contexts. Accordingly, the explanatory variables were categorized into these four levels.

#### Individual-level factors

Individual-level factors comprised personal demographic characteristics, knowledge, reproductive experiences, and health-related behaviors that may be associated with the risk of self-reported sexually transmitted infections (STIs). These variables included age (15–19, 20–24, 25–29, 30–34, 35–39, 40–44, 45–49 years), age at first sex (≤ 19 years, ≥ 20 years), educational attainment (no education, primary, secondary/higher), religion (Christian, Islam, Traditional, no/other religion), comprehensive HIV and AIDS knowledge (no, yes), condom use during last sexual intercourse (no, yes), employment status (not working, working), ever tested for HIV (no, yes), history of terminated pregnancy (no, yes), and number of sexual partners excluding a spouse in the preceding 12 months (zero, one, two or more).

#### Interpersonal-level factors

Interpersonal-level factors reflected relationship and household dynamics that may influence sexual behaviors, decision-making, autonomy, and access to resources. These factors included marital status (never married, married, cohabiting, widowed, divorced) and sex of household head (male, female).

#### Community-level factors

Community-level factors represented contextual characteristics of the immediate social and residential environment that may influence health behaviors and access to information. These variables included mass media exposure (no, yes) and place of residence (urban, rural).

#### Societal/structural-level factors

Societal/structural-level factors captured broader socioeconomic, health-system, and geographic contexts that may shape access to healthcare services, resources, and health opportunities. These variables included wealth index (poorest, poorer, middle, richer, richest), health insurance coverage (no, yes), and sub-region (West Africa, Central Africa, East Africa, Southern Africa).

### Operational definitions

#### Mass media exposure

Mass media exposure was defined by activities such as listening to the radio, watching television, and reading newspapers and magazines. The response options for these three variables were the same: “not at all,” “less than once a week,” and “at least once a week.” Based on the literature [39, 40], we categorized the responses into two groups: “no,” indicating no exposure to mass media (not at all), and “yes,” indicating exposure to mass media (less than once a week and at least once a week).

#### Comprehensive HIV and AIDS knowledge

Comprehensive HIV and AIDS knowledge was measured by asking sexually active women in Africa about their agreement or disagreement with five statements: (a) always use condoms during sex can prevent HIV transmission; (b) a healthy looking person can have HIV; (c) a person can get HIV from mosquito bites; (d) a person can get HIV by sharing food with person who has AIDS; and (e) having one sex partner only, who has no other partners. A composite score was generated based on correct answers to all five statements, with comprehensive HIV and AIDS knowledge classified dichotomously as “Yes” (1) for those who answered all items correctly and “No” (0) for those who provided at least one incorrect response [41, 42].

#### Pregnancy termination

The DHS categorizes pregnancy termination to encompass pregnancy that resulted in miscarriage, induced abortions, or stillbirth. The response options were "Yes" or "No". For this research, the variable, "terminated pregnancy,” was defined as a binary indicator, marked as "Yes" for women who reported having a pregnancy terminated at any point and "No" for those who did not. This methodology aligns with earlier studies utilizing DHS data [43, 44]

### Statistical analysis

**Fig. 1:**
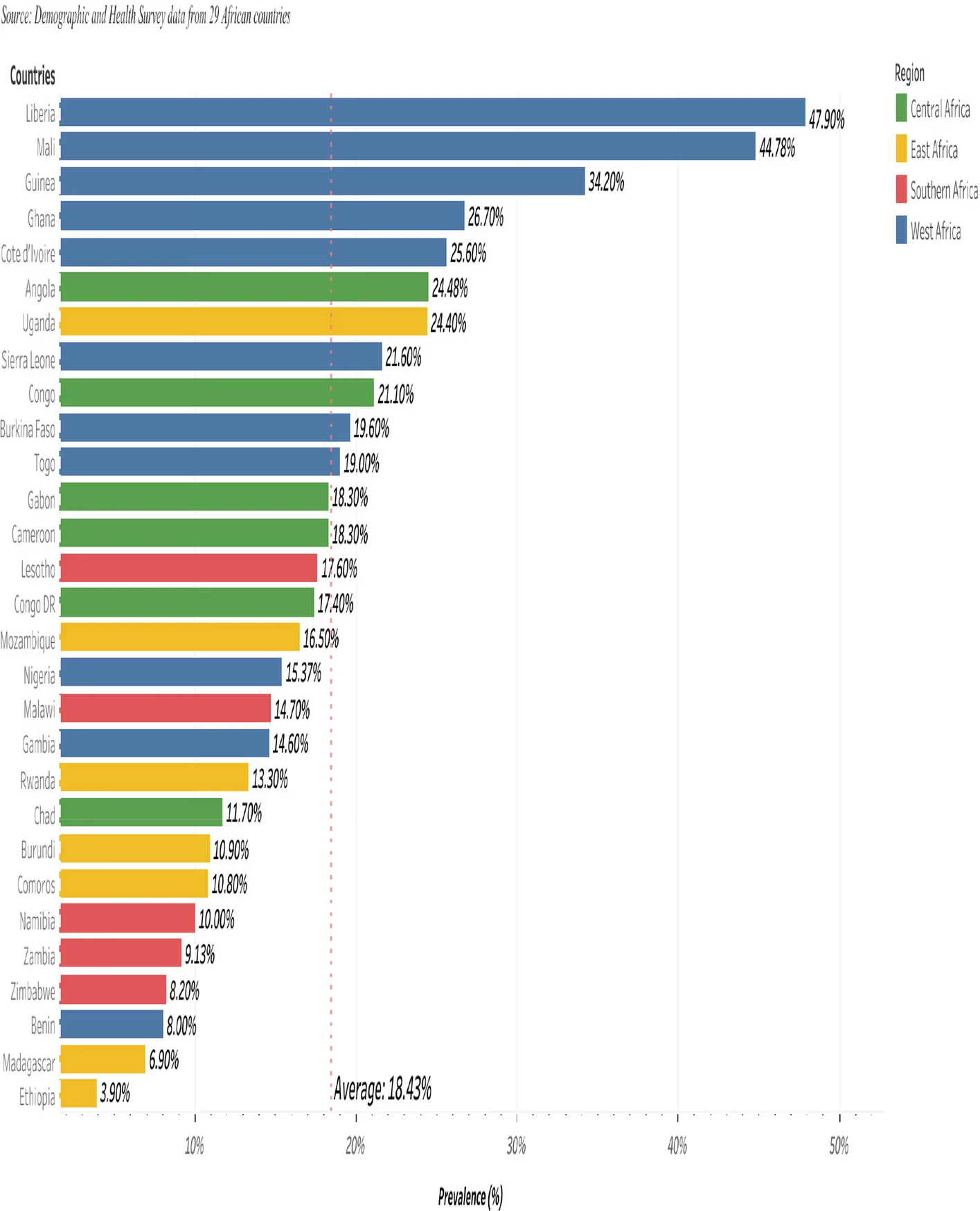
Prevalence (%) of self-reported sexually transmitted infections among women in Africa

Stata version 14.2 was used to analyse the data for this study. Three levels of analysis (univariate, bivariate and multivariate) were carried out. At the univariate level, a graphical representation was used to illustrate the prevalence of SR-STIs across Africa (Fig. 1). In the bivariate analysis, descriptive statistics related to the characteristics of the study and SR-STIs were generated. The prevalence of SR-STIs among sexually active women in Africa was displayed as a bar chart. Subsequently, Table 2 displayed a bivariate analysis of the percentage of SR-STIs across the independent variables. Specifically, the explanatory variables’ weighted frequencies and percentages were reported with p-values from Pearson's chi-square (X^2^). A p-value of less than 0.05 (*p* < 0.05) was used as the criterion for statistical significance (Table 3). Statistically significant variables in the bivariate analysis were moved to the multilevel regression model. Adjusted odds ratios (aORs) with 95% confidence intervals (CIs) were used to present the results of the multilevel regression.

**Table 2:** Socio-demographic characteristics of sexually active women in Africa (Weighted, *N* = 340,179)

Variables	Weighted N	Weighted (%)	χ2 (*p*-value)
Individual-level factors			
Age			**5.72, *p* < 0.001**
15–19	37,383	11.0	
20–24	64,878	19.1	
25–29	65,849	19.4	
30–34	56,701	16.7	
35–39	49,092	14.4	
40–44	37,317	10.9	
45–49	28,959	8.5	
Age at first sex			**891.56, *p* < 0.001**
*≤ 19*	275,530	81.0	
*≥ 20*	64,649	19.0	
Education			**306.01, *p* < 0.001**
No education	110,585	32.5	
Primary	100,819	29.6	
Secondary+	128,775	37.9	
Religion			**6.26, *p* < 0.001**
Christian	218,775	64.3	
Islam	104,205	30.6	
Traditional	4,976	1.5	
No/other	12,223	3.6	
Comprehensive HIV and AIDS knowledge			**1.65, *p* = 0.200**
No	110,834	32.6	
Yes	229,345	67.4	
Condom use during last sex			**158.94, *p* < 0.001**
No	313,787	92.2	
Yes	26,392	7.8	
Employment status			**39.32, *p* < 0.001**
Not working	96,330	28.4	
working	246,225	71.6	
Ever tested for HIV			**122.43, *p* < 0.001**
No	142,125	41.8	
Yes	198,054	58.2	
Terminated Pregnancy			**6.53, *p* < 0.001**
No	283,205	83.2	
Yes	56,974	16.8	
Number of sex partners excluding spouse in the last 12 months			**9.25, *p* < 0.001**
Zero	280,333	82.4	
One	54,047	15.9	
Two or more	5,799	1.7	
Interpersonal-level Factors			
Sex of household head			**16.06, *p* < 0.001**
Male	250,909	73.8	
Female	89,270	26.2	
Marital Status			**820.66, *p* < 0.001**
Never Married	53,466	15.7	
Married	195,163	57.4	
Cohabitation	57,794	17.0	
Widowed	8,824	2.6	
Divorced	24,932	7.3	
Community-level Factors			
Mass Media Exposure			**5.99, *p* < 0.001**
No	124,059	36.5	
Yes	216,120	63.5	
Place of residence			**569.75, *p* < 0.001**
Urban	135,291	39.8	
Rural	204,888	60.2	
Societal-level factors			
Health insurance coverage			**56.19, *p* < 0.001**
No	300,666	88.4	
Yes	39,513	11.6	
Wealth index			**395.47, *p* < 0.001**
Poorest	63,039	18.5	
Poorer	64,926	19.1	
Middle	66,331	19.5	
Richer	70,753	20.8	
Richest	75,130	22.1	
Sub-region			**18.77, *p* < 0.001**
West Africa	143,328	42.2	
Central Africa	61,346	18.0	
East Africa	124,615	36.6	
Southern Africa	10,890	3.2	

*p*-value less than 0.05 indicates statistical significance

**Table 3:** Prevalence of SR-STIs among sexually active women in African

Variables	No (%)	Yes (%)
Individual-level factors		
Age		
15–19	80.9	19.1
20–24	79.5	20.6
25–29	79.9	20.1
30–34	81.1	18.9
35–39	82.4	17.6
40–44	84.4	15.6
45–49	87.0	13.0
Education		
No education	82.6	17.4
Primary	83.1	16.9
Secondary+	79.5	20.5
Age at first sex		
	80.6	19.4
	85.7	14.3
Religion		
Christian	82.9	17.1
Islam	78.2	21.8
Traditional	83.0	17.0
No/other	85.6	14.5
Comprehensive HIV and AIDS knowledge		
No	81.5	18.5
Yes	81.6	18.4
Condom use during last sex		
No	81.9	18.1
Yes	78.2	21.8
Employment status		
Not working	82.3	17.7
working	81.3	18.7
Ever tested for HIV		
No	82.5	17.5
Yes	80.9	19.1
Terminated Pregnancy		
No	82.7	17.3
Yes	75.9	24.1
Number of sex partners excluding spouse in the last 12 months		
Zero	82.9	17.1
One	76.9	23.1
Two or more	62.2	37.8
Interpersonal-level factors		
Marital Status		
Never Married	78.3	21.7
Married	83.0	17.0
Cohabitation	79.2	20.8
Widowed	87.8	12.2
Divorced	81.3	18.7
Sex of household head		
Male	81.5	18.5
Female	81.8	18.2
Community-level Factors		
Mass Media Exposure		
No	85.2	14.8
Yes	79.5	20.1
Place of residence		
Urban	78.9	21.1
Rural	83.4	16.6
Societal-level Factors		
Health insurance coverage		
No	81.7	18.3
Yes	80.5	19.5
Wealth index		
Poorest	84.4	15.6
Poorer	82.6	17.4
Middle	81.7	18.3
Richer	79.8	20.2
Richest	79.9	20.1
Sub-region		
West Africa	76.3	23.7
Central Africa	83.8	19.2
East Africa	87.4	12.6
Southern Africa	89.3	10.7

Multilevel regression analysis was performed to identify the factors associated with SR-STIs among sexually active women in Africa. Multicollinearity was checked among the independent variables using the variance inflation factor (VIF), and the results showed no evidence of high collinearity (mean VIF = 1.25, maximum = 1.76, minimum = 1.04). The highest VIF was observed for wealth quintile, and the lowest for pregnancy termination.

In the multilevel regression, six models (Models 0-V) were constructed (Table 4). The first model (Model 0) was the empty model, which had no explanatory variable. Models I, II, III and IV were based on the components of the socio-ecological model: individual-level factors, interpersonal-level factors, community-level factors, and societal-level factors, respectively. The final model (Model V) was the complete model that contained all the factors. All variables were entered into the final multilevel model simultaneously to allow for mutual adjustment, controlling for potential confounding across all included factors. Model comparison was done using the log-likelihood ratio (LLR) and Akaike's Information Criterion (AIC) tests. The study sample was weighted (v005/1,000,000) and analyzed using Stata’s survey set command (svy) to account for the survey's complexity and the results' generalizability. We restricted our analysis to complete cases; therefore, all missing values were dropped. The extent of missing data was assessed prior to exclusion. Nevertheless, the potential impact of excluding missing cases on the precision of estimates is acknowledged as a limitation.

**Table 4:** Multilevel regression analysis on the factors associated with STIs among sexually active women in Africa

Variables	Null model	Model I	Model II aOR (95% CI)	Model III aOR (95% CI)	Model IV aOR (95% CI)	Model V aOR (95% CI)
Fixed effect						
*Individual-level factors*						
Age						
15–19		Ref				Ref
20–24		1.15*** (1.10–1.20)				1.12** (1.07–1.17)
25–29		1.17*** (1.12–1.22)				1.10** (105–1.15)
30–34		1.10** (1.05–1.15)				1.04 (0.99–1.09)
35–39		1.01 (0.96–1.06)				0.95* (0.90–1.00)
40–44		0.88*** (0.84–0.93)				0.85*** (0.80–0.89)
45–49		0.73*** (0.68–0.77)				0.71* (0.66–0.76)
Education						
No education		Ref				Ref
Primary		1.03 (1.00–1.07)				1.17*** (1.13–1.21)
Secondary+		1.20 (1.15–1.25)				1.09*** (1.05–1.14)
Age at first sex						
*≤ 19*		Ref				Ref
*≥ 20*		0.72*** (0.69–0.75)				0.76*** (0.73–0.79)
Religion						
Christians		Ref				Ref
Islam		1.57*** (1.51–1.64)				1.19*** (1.14–1.25)
Traditional		1.22** (1.06–1.40)				0.93 (0.80–1.05)
No/other		0.87*** (0.80–0.94)				0.92* (0.85–1.00)
Condom use during last sex						
No		Ref				Ref
Yes		0.99 (0.94–1.03)				1.07** (1.02–1.12)
Employment status						
Not working		Ref				Ref
Working		1.16*** (1.12–1.19)				1.07*** (1.04–1.11)
Ever tested for HIV						
No		Ref				Ref
Yes		1.14*** (1.12–1.19)				1.25*** (1.21–1.29)
Terminated Pregnancy						
No		Ref				Ref
Yes		1.56*** (1.51–1.62)				1.46*** (1.41–1.50)
Number of sex partners excluding spouse in the last 12 months						
Zero		Ref				Ref
One		1.49*** (1.43–1.54)				1.62*** (1.54–1.71)
Two or more		2.82*** (2.62–3.04)				3.06***(2.81–3.34)
*Interpersonal-level factors*						
Marital Status						
Never Married			Ref			Ref
Married			0.73*** (0.70–0.76)			1.15*** (1.08–1.22)
Cohabitation			0.94 (0.89–0.98)			1.46*** (1.38–1.56)
Widowed			0.52*** (0.48–0.56)			0.93* (0.85–1.01)
Divorced			0.85** (0.81–0.90)			1.17*** (1.10–1.24)
Sex of household head						
Male			Ref			Ref
Female			0.93*** (0.90–0.96)			0.96** (0.93–0.99)
*Community-level factors*						
Mass media exposure						
No				Ref		Ref
Yes				1.40*** (1.35–1.45)		1.28*** (1.23–1.32)
Place of residence						
Urban				Ref		Ref
Rural				0.81*** (0.78–0.85)		1.04 (0.99–1.10)
*Societal-level factors*						
Wealth index						
Poorest					Ref	Ref
Poorer					1.13*** (1.08–1.19)	1.06* (1.01–1.011)
Middle					1.20** (1.14–1.26)	1.06* (1.01–1.12)
Richer					1.37*** (1.30–1.44)	1.14** (1.08–1.20)
Richest					1.38*** (1.30–1.46)	1.12** (1.05–1.20)
Health insurance coverage						
No					Ref	Ref
Yes					1.07** (1.02–1.12)	1.03** (0.96–1.05)
Sub-region						
Western Africa					Ref	Ref
Central Africa					0.75*** (0.71–0.79)	0.72*** (0.67–0.76)
Eastern Africa					0.46*** (0.44–048)	0.45*** (0.43–0.47)
Southern Africa					0.36*** (0.33–0.40)	0.35*** (0.32–0.39)
Random effect results						
PSU variance (95% CI)	0.08 (0.07–0.10)	0.07 (0.06–0.09)	0.08 (0.07–0.09)	0.08 (0.07–0.09)	0.10 (0.08–0.12)	0.08 (0.06–0.09)
ICC	24.07%	21.59%	23.01%	24.29%	28.34%	22.68%
Wald chi-square	Ref	3353.49**	475.53***	601.25***	1584.07***	5115.56***
Model fitness						
Log likelihood	−161,686.8	−157,644.41	−161,155.62	−160,621.34	−158,338.59	−154,587.00
AIC	323,377.5	315,328.80	322,325.20	321,250.70	316,697.20	309,244.10
BIC	323,399.0	315,543.50	322,400.40	321,293.60	316,804.60	309,619.80
Weighted sample (N)	340,179	340,179	340,179	340,179	340,179	340,179
Number of groups	1,396	1,396	1,396	1,396	1,396	1,396

Source: 2011–2024 DHSs

*Ref* Reference category, *aOR* adjusted odds ratio, *PSU* Primary Sampling Unit, *ICC* Intra-Class Correlation Coefficient, *AIC* Akaike’s Information Criterion, *BIC* Bayesian Information Criterion

^*^p < 0.05, ^**^p < 0.01, ^***^p < 0.001

## Results

### Prevalence (%) of self-reported sexually transmitted infections among women in Africa

The pooled prevalence of SR-STIs among sexually active women in Africa was 18.43% [95% CI: 18.00%–18.70%]. Although the pooled estimate shows high statistical precision due to the large sample size, substantial heterogeneity existed across countries, with prevalence ranging from 3.9% in Ethiopia to 47.9% in Liberia (Fig. 1).

### Socio-demographic characteristics of sexually active women in Africa

Table 2 presents the socio-demographic characteristics of sexually active women in Africa, with the majority of participants aged 25–29 years (19.4%), had secondary and higher education (37.9%), were married (57.4%), were employed (71.6%), were not covered by health insurance (88.4%), had a history of terminated pregnancy (83.2%), had no sex partners excluding spouse in the last 12 months (82.4%), had ever been tested for HIV (58.2%), had a comprehensive HIV and AIDS knowledge (67.4%), had first sex at age ≤ 19 years (81.0%), and did not use a condom during their last sexual encounter (92.2%). Over half of the respondents (63.5%) had been exposed to mass media, while 64.3% identified as Christians, 73.8% had male heads of household, 22.1% belonged to the wealthiest quintile, 60.2% lived in rural areas, and 42.2% resided in West Africa, respectively.

The analysis indicated that factors such as age, education, marital status, employment status, health insurance coverage, terminated pregnancy, number of sexual partners excluding spouse in the 12 months, ever tested for HIV, condom use during last sex, age at first sex, mass media exposure, religion, sex of household head, wealth index, place of residence, and sub-region were statistically significantly associated with SR-STIs among sexually active women in Africa (Table 2).

### Prevalence of SR-STIs among sexually active women in African

In Table 3, the highest prevalence of SR-STIs was reported among those aged 20–24 years (20.6%), with secondary or higher education (20.5%), those never married (21.7%), those working (18.7%), and those covered by health insurance (19.5%). Regarding terminated pregnancy, women with a history of pregnancy termination (24.1%), had two or more sex partners excluding spouse in the last 12 months (37.8%), had ever been tested for HIV (19.1%), used a condom during last sex (21.8%), and had sex at age 19 or less (19.4%) had the highest proportion of SR-STIs. The highest proportion of SR-STIs was observed among those exposed to mass media (20.5%), Muslims (21.8%), male household heads (18.5%), individuals in the wealthiest quintile (20.2%), residents of urban areas (21.1%), and women in West Africa (23.7%).

### Multilevel regression analysis on the factors associated with STIs among sexually active women in Africa

Table 4 presents the multilevel regression results. The final model (Model V), which contains all the independent variables. However, at the individual level, higher odds of SR-STIs were observed among women with primary education [aOR = 1.17; 95% CI = 1.13–1.21], Muslim women [aOR = 1.19; 95% CI = 1.14–1.25], women who used condoms during their last sex [aOR = 1.07; 95% CI = 1.02–1.12], working women [aOR = 1.07; 95% CI = 1.04–1.11], ever terminated a pregnancy [aOR = 1.46; 95% CI = 1.41–1.50], women who had ever tested for HIV [aOR = 1.25; 95% CI = 1.21–1.29] and women with two or more sexual partners excluding spouses were more likely to self-report STIs [aOR = 3.06; 95% CI = 2.81–3.34] compared to those who had no formal education, Christians, never used condoms during their last sex, not working, to those who had never terminated a pregnancy and never tested for HIV, and those who had no other sexual partner., respectively.

At the interpersonal-level, the likelihood of SR-STIs was higher among sexually active women who were cohabiting [aOR = 1.46; 95% CI = 1.38–1.56] compared to those who were never married while at the community level the likelihood of SR-STIs was higher among sexually active women who were exposed to mass media [aOR = 1.28; 95% CI = 1.23–1.32] compared to those who were exposed to mass media.

At the societal-level, higher odds of SR-STIs were observed among sexually active women who were in the richest wealth index [aOR = 1.12; 95% CI = 1.05–1.20] and covered by health insurance [aOR = 1.03; 95% CI = 0.96–1.05] compared to those who in the poorest wealth index and not covered by health insurance, respectively.

At the individual level, the likelihood of SR-STIs was lower among women aged 45–49 [aOR = 0.71; 95% CI = 0.66–0.76] and women whose first sexual encounter occurred at age 20 or older [aOR = 0.76, 95% CI = 0.73–0.79] compared with those aged 15–19, and who had their first sex below age 20. At the interpersonal level, female household heads [aOR = 0.96, 95% CI = 0.93–0.99], and at the societal-level, women in Southern Africa had a lower likelihood of self-reporting STIs [aOR = 0.35; 95% CI = 0.32–0.39] compared to those in male household heads compared to those in West Africa, respectively.

### Random effects (variance measures) results

As shown in Table 4, the AIC values decreased progressively from the null model to Model V, indicating that Model V had the best fit. This substantial decrease in the models supports the goodness of fit of model V, which had all the explanatory variables. Model V is the complete model that was selected for predicting the factors associated with SR-STIs among sexually active women in Africa. In the empty model, substantial variations were observed in the likelihood of factors associated with SR-STIs among sexually active women across the clustering of the primary sampling units (PSUs) [σ2 = 0.08, 95% CI 0.07–0.10]. The ICC value for the null model shows that 24.07% of the variation in the SR-STIs among sexually active women was attributed to the between-cluster variations of the characteristics. The variation between clusters then decreased to 21.59% in Model I, which was the individual-level factors. The ICC then increased to 23.01% in Model II, 24.29% in Model III, 28.34% in Model IV which had only the interpersonal-level, community-level and societal-level factors, respectively. In the final model (Model V), the between-cluster variation decreased to 22.68%. This can be attributed to the variations in how the PSUs are clustered, which influence the differences in SR-STIs among sexually active women in Africa.

## Discussion

Sexual and reproductive health are significantly impacted by STIs [45, 46]. The WHO has supported countries to develop national strategic plans and guidelines that will not only create an enabling environment allowing individuals to discuss STIs, adopt safer sexual practices, and seek treatment, but also strengthen capacity to monitor STI trends, enhance and scale up health intervention and primary prevention, and facilitate the adoption of point-of-care tests [47]. This study investigated the prevalence of, and factors associated with STIs among sexually active women in Africa, based on the most recent DHS dataset.

This study estimated the prevalence of SR-STIs among sexually active women in Africa to be 18.43%. This finding highlights a critical public health concern with important implications for women's sexual and reproductive health and broader societal impacts. Compared with other LMICs, Africa exhibits the highest prevalence of STIs, largely attributable to limited sexual health resources and inadequate healthcare infrastructure [47, 48]. The observed prevalence of SR-STIs highlights the need to strengthen programs and policies across Africa to ensure sustained, affordable access to STI prevention and treatment services [9, 45]. Compared with previous studies, the prevalence reported in our study is higher than the 11.6% found among reproductive-age women in Southeast Asia [49], 6.92% among adolescent girls in SSA [9], and 5.8% among either married or cohabiting women in SSA [3]. However, it is lower than the prevalence of 28.0% in Uganda [50], 19.4% in Eswatini [2], and 14.1% in Mali [51]. The variations in prevalence could be attributable to the differences in sexual behaviour, sociocultural norms, access to STI screening and treatment services, and the reliance on the self-reported data [9, 52].

The study found a regional variation in the prevalence of STIs across Africa, with Liberia, a country in West Africa, having the highest prevalence of 47.9%, while the least was reported in Ethiopia, a country in East Africa, with a prevalence of 3.9%. To reduce the high prevalence of STIs, coordinated programmes and policies have been implemented in Liberia through a partnership between the government, its agencies, and non-governmental organizations. Among these are the national dual HIV/syphilis rapid tests and strategic allocation of resources directly to county and district health teams, which is crucial for ensuring service quality and comprehensiveness [53].

However, at the sub-regional level, our results revealed that sexually active women from Southern Africa had lower odds of SR-STIs. This finding is noteworthy given that Southern Africa bears the highest global burden of HIV [54]. Several factors may explain this paradox. Possible explanations include the intensive HIV prevention investments (e.g., PEPFAR, Global Fund) that integrated STI services, higher health-seeking behavior and treatment coverage, reducing infectious periods; regional differences in sexual networks and condom use; and stronger health system infrastructure.

The regional variation in the prevalence of STIs across Africa calls for the implementation of improved measures by the respective subregions towards mitigating the high prevalence of STIs on the continent. We therefore advocate for comprehensive STI education and promotion as an effective means of reducing the burden of STIs in Africa. For instance, China's comprehensive approach to STI management, bolstered by a loan from the World Bank and executed through community-based collaborations, led to notable advancements in knowledge, behavioral change, and prevalence rates [55]. Specifically, in areas where the project was implemented from 2002 to 2007, the percentage of the general population providing correct answers to HIV/AIDS/STI knowledge questions increased from 3.1% to 17.4%, and condom use during the most recent sexual activity increased from 19.8% to 30.1% [55].

Using the SEM levels, several variables are either risk or protective factors associated with SR-STIs among sexually active women in Africa. These risk factors are education, condom use during last sex, mass media exposure, employment status, marital status, religion, wealth quintile, health insurance coverage, terminated pregnancy, ever tested for HIV, and the number of sex partners excluding spouses in the last 12 months. However, age, sex of household head, age at first sex, and subregion were protective factors.

This study found that the prevalence of SR-STIs decreased with increasing age, consistent with findings from Eswatini [2] and Malawi [56]. Several biological and behavioral mechanisms help explain why women aged 20–29 years carry a higher burden of STIs. The biological susceptibility of women aged 20–29 years to STIs cannot be overemphasized and therefore requires stringent measures to curb sexual exploitation [57, 58]. Women aged 20–29 years are often more likely to participate in sexual behaviors associated with increased STI acquisition risk, including early sexual initiation, relationships with significantly older partners, and inconsistent condom use, typically in situations where power imbalances hinder their ability to negotiate safer sex [9, 59–61]. Structurally, younger women may have limited access to comprehensive sexuality education and youth-friendly health services, which can reduce awareness of STI symptoms and preventive practices, contributing to delayed care-seeking and continued transmission [62, 63].

Education is widely recognized as a factor that enhances women’s autonomy, understanding of health issues, and access to healthcare services [64]. Consistent with previous studies, we found a significant association between education level and SR-STIs [64–66]. We found that sexually active women who had at least a formal education had higher odds of SR-STIs compared to those without formal education. This outcome does not necessarily suggest that educated women face a greater biological risk; rather, it may indicate a heightened awareness of STI symptoms, better health-seeking behaviors, and an increased tendency to identify and report infections. Women with formal education are more inclined to access information regarding sexual and reproductive health, get tested, and utilize health services, thereby enhancing the likelihood of SR-STIs. Systematic reviews have documented similar trends, revealing that education enhances knowledge and reporting of sexual health issues without necessarily leading to an increase in risky sexual practices [67, 68]. Conversely, women with limited or no education may experience underdiagnosis and underreporting due to a lack of awareness, stigma, and decreased access to healthcare, which conceals the actual incidence of STIs in this demographic [69].

In line with research conducted in Africa [2, 69, 70] and Indonesia [71], sexually active women who were employed reported higher incidences of SR-STIs compared to those who were unemployed. A plausible reason could be that employed women tend to have better access to health information, screenings for STIs, and healthcare services due to financial resources or benefits related to their jobs, which enhances their ability to recognize symptoms and increases the chances of testing and receiving a diagnosis [69].

Consistent with a previous study in Africa [72], sexually active women who were cohabiting had higher odds of SR-STIs. A possible explanation is that cohabiting women may engage in unprotected sex with partners who have multiple sexual partners and may also lack the autonomy to negotiate safer sex, thereby increasing their risk of acquiring STIs and, consequently, their likelihood of self-reporting STIs [73, 74].

Similar to previous studies conducted in Africa [2, 9, 35, 50], this current study found that sexually active women with two or more sexual partners excluding their spouses were more likely to have higher odds of SR-STIs compared with those who had no sexual partners aside from their spouse in the last 12 months. Possible reasons include a lack of communication and romantic intimacy, dissatisfaction with a partner's sexuality, or inadequate sexual skills [56]. To mitigate SR-STI risk in this population, focused interventions should emphasize comprehensive sexual health education, the promotion of regular condom use, and routine STI screenings, while also addressing gender power dynamics and enhancing access to women-centered sexual and reproductive health services.

Consistent with studies conducted in China [75], South Africa [76], and the United States of America (USA) [77], this study found that women who had ever terminated a pregnancy had higher odds of SR-STIs. Closely related is religion, our study found that sexually active Muslim women had higher odds of SR-STIs. This finding is consistent with a country-level study in Uganda [50]. A possible explanation for this could be attributed to the widespread practice of polygamy [50].

In this study, sexually active women in Africa who have subscribed to health insurance had higher odds of SR-STIs. This finding is similar to another study in SSA [69, 78]. However, this finding should be interpreted cautiously, as it is likely influenced by reverse causality and differential detection rather than increased biological risk. One potential reason for this is that these sexually active women might have better financial means to obtain STI screening and testing [69]. Furthermore, a higher socioeconomic status is frequently linked to increased opportunities for travel for work or leisure and enhanced involvement in social networks, which could lead to more chances for multiple sexual relationships and, as a result, a higher likelihood of reporting STI symptoms [77]. Consequently, uninsured women may experience underdiagnosis and underreporting due to financial and access barriers, potentially masking the true burden of SR-STIs in this group. These findings underscore the importance of strengthening equitable access to STI screening and prevention services, particularly among women without health insurance coverage. Additionally, ever being tested for HIV was associated with SR-STIs, which is consistent with a study conducted in Sierra Leone [78].

Sexually active women in Africa who used condoms during their last sex had higher odds of SR-STIs compared to those who had not used condoms during their last sex. Similar associations have been reported in studies conducted in SSA [35], Europe [79], and Ghana [80]. However, this should not be interpreted as condom ineffectiveness. This association likely reflects reverse causality, incorrect condom use, and STI testing among condom users. Substantial evidence confirms that consistent and correct condom use remains highly effective in preventing STIs [81, 82].

Research consistently shows that starting sexual activity later is linked to a lower risk of STIs among women [83, 84]. Several studies conducted in Africa [83, 85] and Korea [85] have supported this finding that the odds of SR-STIs were lower for sexually active women who had their first sex after age 19. The plausible reason could be that women aged 20 years and older may not engage in sexual practices that increase transmission risk in Africa [86]. Therefore, initiatives that successfully delay sexual initiation among young people are logical components of a broader approach to decreasing sexually transmitted infections (STIs) among adolescents, who are significantly affected by the STI epidemic [85].

As confirmed by studies in SSA [9, 35] and Nigeria [87], this study found that sexually active women in Africa exposed to mass media had higher odds of SR-STIs. Mass media exposure has proven to be one of the main tools for ensuring comprehensive education and the dissemination of vital information when it comes to sexuality education [35]. Another possible reason could be that some mass media outlets, including newspapers and television, may promote content that includes sexually risky behaviours which could influence sexual decision-making in certain contexts [9]. Overall, these findings highlight mass media as a powerful platform for STI prevention while underscoring the need for accurate and evidence-based sexuality education messaging.

In studies conducted in Africa [9, 78], women in the richer and richest wealth quintiles were found to have increased odds of SR-STIs compared with those in the poorest wealth quintile. One potential reason for this is that these sexually active women might have better financial means to obtain STI screening and testing [78]. Furthermore, a higher socioeconomic status is frequently linked to increased opportunities for travel for work or leisure and enhanced involvement in social networks, which could lead to more chances for multiple sexual relationships and, as a result, a higher likelihood of reporting STI symptoms [88]. This finding is the direct opposite of studies conducted in Bangladesh [89] and the USA [90], where poorer participants were found to have higher odds of STIs.

In conclusion, the regionally diverse prevalence of SR-STIs among sexually active women in Africa highlights the critical necessity for tailored, gender-sensitive, and equity-focused strategies that tackle predisposing, enabling, and needs factors while enhancing health systems, broadening fair access to screening and treatment, and utilizing education and mass media for effective sexual health advocacy.

### Strengths and limitations of the study

This research used the most recent datasets from DHS in Africa, which incorporate strong and well-established methods for data collection and have been extensively employed in prior studies. Another strength is the robust analytical and statistical methodology applied to enhance the reliability of the results. However, several limitations should be acknowledged. First, the cross-sectional and secondary nature of the DHS data restricts the ability to establish causality and raises the possibility of reverse causation, as the analysis was confined to women's data files. This is particularly pertinent for variables such as condom use during last sex, HIV testing history, health insurance coverage, and mass media exposure. For instance, women who experienced STI symptoms may have subsequently sought HIV testing, used condoms, or accessed health information through media, rather than these factors increasing their risk of STIs. Thus, these associations may reflect reverse causality or detection bias rather than causal effects and should be interpreted with caution. Second, although a large sample size was employed, the results may not be generalizable to all sexually active women in Africa. Consequently, the outcomes of this research should be viewed with caution. Additionally, care should be taken when interpreting the odds, as we recognize that the higher odds ratios related to some variables might be due to the substantial sample size used in this study.

Third, the DHS does not provide laboratory-confirmed STI diagnoses or details regarding treatment history of STIs, which may result in misclassification and hinder differentiation between current, past, and recurring infections. In particular, the reliance on self-reported symptoms and diagnoses may lead to an overestimation of symptomatic infections while simultaneously underestimating the true burden of asymptomatic STIs, which are common among women.

Fourth, factors related to health systems, including STI screening availability, surveillance systems, and national sexual health policies, were also not directly considered and could contribute to differences across sub-regions. Finally, although multilevel modelling addressed clustering at the PSU level, residual community-level confounding related to cultural norms and gender dynamics may still exist. Despite these limitations, this study provides valuable, multi-country evidence on the prevalence and multilevel factors associated with SR-STIs among sexually active women in Africa, offering important insights for targeted interventions and policy development.

## Conclusion and recommendation

This research offers extensive, cross-country insights into the prevalence and various factors influencing SR-STIs among sexually active women in Africa. The research revealed that the prevalence of SR-STIs among sexually active women in Africa was high at 18.43%. There were significant differences between countries, with rates ranging from 47.9% in Liberia to 3.9% in Ethiopia, underscoring the uneven distribution of SR-STIs across Africa. The results align with socio-ecological model, suggesting that SR-STIs are shaped by a complex interplay of individual, interpersonal, community and societal factors.

Major risk factors for SR-STIs include higher education levels, cohabitation, exposure to mass media, working, condom use during the last sex, history of pregnancy termination, ever being tested for HIV, having multiple sexual partners excluding spouse in the last 12 months, being Muslim, being in the richer or richest wealth quintile, and being covered by health insurance. Conversely, older women, late initiation of sexual activity, living in female-led households, and Southern Africa were found to be protective factors.

Addressing the significant and uneven prevalence of SR-STIs among sexually active women in Africa necessitates focused, contextually appropriate strategies that are guided by the multilevel factors identified in this research. Programs implemented by various governments in Africa should incorporate STI education and routine screening into standard reproductive health and HIV services. Also, there is the need for coordinated programs and policies that will increase health insurance coverage and improve access to quality care. Considering the significant disparities between countries, region-specific approaches that reflect contextual and health system variations are crucial. Tackling these multilevel factors is crucial for promoting reproductive health and accelerating progress towards broader sexual and reproductive health objectives in the region.

## Data Availability

The dataset used is freely available at (https://dhsprogram.com/data/available-datasets.cfm).

## Abbreviations

AIC: Akaike’s Information Criterion
aOR: Adjusted odds ratio
CI: Confidence intervals
DHS: Demographic and health Survey
ICC: Intra-Class Correlation Coeficient
LMICs: Low-and-middle-income countries
MMR: Maternal Mortality Ratio
PIDs: Pelvic inflammatory disease
PSU: Primary Sampling Unit
Ref: Reference category
SR: Self reporting
SSA: Sub-Saharan Africa
STIs: Sexually Transmitted Infections
UHC: Universal Health coverage
VIF: Variance Inflation Factor
WHO: World Health Organization

## References

[1] World Health Organization. Implementing the global health sector strategies on HIV, viral hepatitis and sexually transmitted infections, 2022–2030: report on progress and gaps 2024. World Health Organization; 2024.

[2] Ginindza TG, Stefan CD, Tsoka-Gwegweni JM, Dlamini X, Jolly PE, Weiderpass E, Sartorius B. Prevalence and risk factors associated with sexually transmitted infections (STIs) among women of reproductive age in Swaziland. Infect Agent Cancer. 2017;12:1–12. 10.1186/s13027-017-0140-y PMC5445272 28559923

[3] Adu C, Mohammed A, Budu E, Frimpong JB, Tetteh JK, Ahinkorah BO, et al. Sexual autonomy and self-reported sexually transmitted infections among women in sexual unions. Arch Public Health. 2022;80(1):40. 10.1186/s13690-022-00796-4 PMC8790891 35081983

[4] Gottlieb SL, Spielman E, Abu-Raddad L, Aderoba AK, Bachmann LH, Blondeel K, Broutet N. WHO global research priorities for sexually transmitted infections. Lancet Glob Health. 2024;12(9):e1544-e1551. 10.1016/S2214-109X(24)00266-3 PMC11342064 39043199

[5] Ong JJ, Unemo M, Molina JM, Miranda AE, Seib KL, Brito de Mello M, Doherty M, Johnson CC, Delany-Moretlwe S, & Fairley C. Beyond behavioural change: prioritising structural solutions to control bacterial sexually transmitted infections. E Clin Med. 2025;83:103198. 10.1016/j.eclinm.2025.103198Ong. PMC12235396 40630608

[6] World Health Organization. Sexually transmitted infections (STIs) fact sheet. 2024. Available from: https://www.who.int/news-room/fact-sheets/detail/sexually-transmitted-infections-(stis). Cited 2024 Jul 8.

[7] World Health Organization. Global health sector strategies on, respectively, HIV, viral hepatitis and sexually transmitted infections for the period 2022-2030. World Health Organization; 2022.

[8] Zamora G, Koller TS, Thomas R, Manandhar M, Lustigova E, Diop A, et al. Tools and approaches to operationalize the commitment to equity, gender and human rights: towards leaving no one behind in the Sustainable Development Goals. Glob Health Action. 2018;11(sup1):75–81. 10.1080/16549716.2018.1463657 PMC5974708 29808773

[9] Dadzie LK, Agbaglo E, Okyere J, Aboagye RG, Arthur-Holmes F, Seidu AA, et al. Self-reported sexually transmitted infections among adolescent girls and young women in sub-Saharan Africa. Int Health. 2022;14(6):545–53. 10.1093/inthealth/ihab088 PMC9623488 35134172

[10] Newman DR, Rahman MM, Brantley A, Peterman TA. Rates of new human immunodeficiency virus (HIV) diagnoses after reported sexually transmitted infection in women in Louisiana, 2000–2015: implications for HIV prevention. Clin Infect Dis. 2020;70(6):1115–20. 10.1093/cid/ciz303 PMC6790153 30976788

[11] Elendu C, Amaechi DC, Elendu ID, Elendu TC, Amaechi EC, Usoro EU, Balogun BB. Global perspectives on the burden of sexually transmitted diseases: A narrative review. Medicine. 2024;103(20):e38199. 10.1097/MD.0000000000038199 PMC11098264 38758874

[12] Torrone EA, Morrison CS, Chen PL, Kwok C, Francis SC, Hayes RJ, STIMA Working Group. Prevalence of sexually transmitted infections and bacterial vaginosis among women in sub-Saharan Africa: an individual participant data meta-analysis of 18 HIV prevention studies. PLoS Med. 2018;5(2):e1002511. 10.1371/journal.pmed.1002511 PMC5828349 29485986

[13] Karim QA, Baxter C, Birx D. Prevention of HIV in adolescent girls and young women: key to an AIDS-free generation. JAIDS J Acquir Immune Defic Syndr. 2017;75:S17–26. 10.1097/QAI.0000000000001316 28398993

[14] Panchanadeswaran S, Johnson SC, Mayer KH, Srikrishnan AK, Sivaran S, Zelaya CE, Celentano DD. Gender differences in the prevalence of sexually transmitted infections and genital symptoms in an urban setting in southern India. Sex Transm Infect. 2006;82(6):491–495. 10.1136/sti.2006.020768 PMC2563883 16757513

[15] Coombs RW, Reichelderfer PS, Landay AL. Recent observations on HIV type-1 infection in the genital tract of men and women. AIDS. 2003;17(4):455–80. 10.1097/00002030-200303070-00001 12598766

[16] Seloka MA, Phalane E, & Phaswana-Mafuya RN. The control and prevention of sexually transmitted infections in primary healthcare facilities among key and priority population in sub-Saharan Africa: A systematic review. African J Reprod Health. 2025;29(3):168–203. https://doi-org.proxy.lib.uwaterloo.ca/10.29063/ajrh2025/v29i3.19. 10.29063/ajrh2025/v29i3.19 40135496

[17] Kisa S, Taskin L. Validity of the symptomatic approach used by nurses in diagnosing vaginal infections. J Clin Nurs. 2009;18(7):1059–68. 10.1111/j.1365-2702.2008.02702.x 19284438

[18] Garrett NJ, Osman F, Maharaj B, Naicker N, Gibbs A, Norman E, Mindel A. Beyond syndromic management: opportunities for diagnosis-based treatment of sexually transmitted infections in low-and middle-income countries. PloS One. 2018;13(4):e0196209. 10.1371/journal.pone.0196209 PMC5918163 29689080

[19] Menezes JRD, Sardinha JCG, Galbán E, Saraceni V, Talhari C. Effectiveness of syndromic management for male patients with urethral discharge symptoms in Amazonas, Brazil. An Bras Dermatol. 2017;92:779–84. 10.1590/abd1806-4841.20175453 PMC5786390 29364432

[20] Redwood-Campbell L, Plumb J. The syndromic approach to treatment of sexually transmitted diseases in low-income countries: issues, challenges, and future directions. J Obstet Gynaecol Can. 2002;24(5):417–24. 10.1016/s1701-2163(16)30405-4 12196862

[21] Stewart J, Bukusi E, Celum C, Delany-Moretlwe S, Baeten JM. Sexually transmitted infections among African women: an underrecognized epidemic and an opportunity for combination STI/HIV prevention. AIDS (London, England). 2020;34(5):651. 10.1097/QAD.0000000000002472 PMC7290066 32167988

[22] Hasani S, Aung E, Mirghafourvand M. Low self-esteem is related to depression and anxiety during recovery from an ectopic pregnancy. BMC Womens Health. 2021;21:1–8. 10.1186/s12905-021-01467-2 PMC8424942 34496785

[23] Aduloju PO, Olagbuji NB, Olofinbiyi AB, Awoleke JO. Prevalence and predictors of intimate partner violence among women attending infertility clinic in south-western Nigeria. Eur J Obstet Gynecol Reprod Biol. 2015;188:66–9. 10.1016/j.ejogrb.2015.02.027 25794827

[24] Stellar C, Garcia-Moreno C, Temmerman M, Van Der Poel S. A systematic review and narrative report of the relationship between infertility, subfertility, and intimate partner violence. Int J Gynecol Obstet. 2016;133(1):3–8. 10.1016/j.ijgo.2015.08.012 26797197

[25] Klausner JD, & Allan-Blitz L-T. The Impacts and Consequences of Sexually Transmitted Infections in the United States. Sex Transm Dis. 2024;52(5):285–289. 10.1097/OLQ.0000000000002126Allan-Blitz PMC11991881 39714030

[26] Abdul R, Gerritsen AA, Mwangome M, Geubbels E. Prevalence of self-reported symptoms of sexually transmitted infections, knowledge and sexual behaviour among youth in semi-rural Tanzania in the period of adolescent friendly health services strategy implementation. BMC Infect Dis. 2018;18:1–10. 10.1186/s12879-018-3138-1 PMC5960083 29778101

[27] Baral S, Logie CH, Grosso A, Wirtz AL, Beyrer C. Modified social ecological model: a tool to guide the assessment of the risks and risk contexts of HIV epidemics. BMC Public Health. 2013;13(1):482. 10.1186/1471-2458-13-482 PMC3674938 23679953

[28] Bull SS, Shlay JC. Promoting “dual protection” from pregnancy and sexually transmitted disease: a social ecological approach. Health Promot Pract. 2005;6(1):72–80. 10.1177/1524839903259308 15574531

[29] Hernandez CJ, Trujillo D, Tate M, Baguso G, Quintana J, McNaughten KC, Wilson EC. Assessing the Risk of Sexually Transmitted Infections among Men who have Sex with Men in San Francisco during COVID-19: A Socio-ecological Analysis. AIDS Behav. 2025;29(4):1352–1361. 10.1007/s10461-024-04608-4 PMC13292803 39798027

[30] Fahme SA, Chehab S, Logie CH, Mumtaz G, Fitzgerald D, Downs JA, Sieverding M. Intersecting social-ecological vulnerabilities to and lived experiences of sexually transmitted infections among Syrian refugee women in Lebanon: A qualitative study. PLOS Glob Public Health. 2024;4(8):e0003507. 10.1371/journal.pgph.0003507 PMC11309427 39116144

[31] DiClemente RJ, Salazar LF, Crosby RA, Rosenthal SL. Prevention and control of sexually transmitted infections among adolescents: the importance of a socio-ecological perspective—a commentary. Public Health. 2005;119(9):825–36. 10.1016/j.puhe.2004.10.015 15913678

[32] Armah-Ansah EK, Bawa B, Dindas J, Budu E, Ahinkorah BO, Ameyaw EK. A multilevel analysis of social determinants of skilled birth attendant utilisation among married and cohabiting women of Madagascar. Int Health. 2023:ihad108. 10.1093/inthealth/ihad108. PMC11532666 38011796

[33] Aliaga A, Ruilin R. Cluster optimal sample size for demographic and health surveys. In 7th International Conference on Teaching Statistics–ICOTS 2006 (Vol. 7, pp. 2–7).

[34] Von Elm E, Altman DG, Egger M, Pocock SJ, Gøtzsche PC, Vandenbroucke JP. The Strengthening the Reporting of Observational Studies in Epidemiology (STROBE) statement: guidelines for reporting observational studies. The lancet. 2007;370(9596):1453–7. 10.1016/S0140-6736(07)61602-X 18064739

[35] Seidu AA, Ahinkorah BO, Dadzie LK, Tetteh JK, Agbaglo E, Okyere J, Schack T. A multi-country cross-sectional study of self-reported sexually transmitted infections among sexually active men in sub-Saharan Africa. BMC Public Health. 2020;20:1–11. 10.1186/s12889-020-09996-5 PMC7722450 33287785

[36] Seidu AA, Agbaglo E, Dadzie LK, Tetteh JK, Ahinkorah BO. Self-reported sexually transmitted infections among sexually active men in Ghana. BMC Public Health. 2021;21(1):993. 10.1186/s12889-021-11030-1 PMC8157633 34039317

[37] Dagnew GW, Asresie MB, Fekadu GA. Factors associated with sexually transmitted infections among sexually active men in Ethiopia. Further analysis of 2016 Ethiopian demographic and health survey data. PLoS ONE. 2020;15(5):e0232793. 10.1371/journal.pone.0232793 PMC7205475 32380513

[38] Masanja V, Wafula ST, Ssekamatte T, Isunju JB, Mugambe RK, Van Hal G. Trends and correlates of sexually transmitted infections among sexually active Ugandan female youths: evidence from three demographic and health surveys, 2006–2016. BMC Infect Dis. 2021;21(1):59. 10.1186/s12879-020-05732-x. PMC7805221 33435882

[39] Bolarinwa OA, Ahinkorah BO, Arthur-Holmes F, Aboagye RG, Kwabena Ameyaw E, Budu E, Yaya S. Sex inequality in early initiation of breastfeeding in 24 sub-Saharan African countries: A multi-country analysis of Demographic and Health Surveys. Plos One. 2022;17(5):e0267703. 10.1371/journal.pone.0267703 PMC9119560 35587942

[40] Sen KK, Mallick TS, Bari W. Gender inequality in early initiation of breastfeeding in Bangladesh: a trend analysis. Int Breastfeed J. 2020;15(1):1–11., 24 10.1186/s13006-020-00259-y PMC7075039 32178697

[41] Darteh EK. Individual and contextual predictors of comprehensive HIV and AIDS knowledge among young females in Ghana. Afr J AIDS Res. 2020;19(3):222–30. 10.2989/16085906.2020.1802307 32892711

[42] Tetteh JK, Frimpong JB, Budu E, Adu C, Mohammed A, Ahinkorah BO, et al. Comprehensive HIV/AIDS knowledge and HIV testing among men in sub-Saharan Africa: a multilevel modelling. J Biosoc Sci. 2022;54(6):975–90. 10.1017/S0021932021000560 34736542

[43] Ba DM, Zhang Y, Pasha-Razzak O, Khunsriraksakul C, Maiga M, Chinchilli VM, et al. Factors associated with pregnancy termination in women of childbearing age in 36 low-and middle-income countries. PLoS Glob Public Health. 2023;3(2):e0001509. 10.1371/journal.pgph.0001509 PMC10021843 36963033

[44] Thabrew KAS, Ranawaka RACS, Ranamukhaarachchi S. Factors associated with terminated pregnancies in Sri Lanka: a case study of the Sri Lankan demographic and health survey (DHS) 2016. PLoS ONE. 2024;19(2):e0298639. 10.1371/journal.pone.0298639 PMC10889877 38394081

[45] World Health Organization (2022). Global health Sector strategies on, respectively, HIV, viral hepatitis and sexually transmitted infections for the period 2022–2030. https://www.who.int/news-room/fact-sheets/detail/sexually-transmitted-infections-(stis)#:~:text=STIs%20have%20a%20profound%20impact,and%20trichomoniasis%20(156%20million). Accessed on 21st July, 2024.

[46] Chesson HW, Mayaud P, Aral SO. Sexually transmitted infections: impact and cost-effectiveness of prevention. In: Major infectious diseases. 3rd edition. Washington (DC): The International Bank for Reconstruction and Development/The World Bank; 2017. 30212101

[47] Taylor MM, Wi T, Gerbase A, Thwin SS, Gottlieb S, Babovic MT, Broutet N. Assessment of country implementation of the WHO global health sector strategy on sexually transmitted infections (2016–2021). PLoS One. 2022;17(5):e0263550. 10.1371/journal.pone.0263550 PMC9067912 35507535

[48] Van Gerwen OT, Muzny CA, Marrazzo JM. Sexually transmitted infections and female reproductive health. Nat Microbiol. 2022;7(8):1116–26. 10.1038/s41564-022-01177-x PMC9362696 35918418

[49] Yamani LN, Astutik E, Qurniyawati E, Lusida MI, Getaneh Y, Kelly M. Associations between socio-demographics, sexual knowledge and behaviour and sexually transmitted infections among reproductive-age women in Southeast Asia: Demographic Health Survey results. BMC Public Health. 2025;25(1):738. 10.1186/s12889-025-21962-7 PMC11847402 39987067

[50] Nankinga O, Misinde C, Kwagala B. Gender relations, sexual behaviour, and risk of contracting sexually transmitted infections among women in union in Uganda. BMC Public Health. 2016;16(1):440. 10.1186/s12889-016-3103-0 PMC4881206 27229928

[51] Appiah CK, Dowou RK, Balame SK, Adzigbli LA, Yeboah PA, Aboagye RG, et al. Self-reported sexually transmitted infections among adolescent girls and young women in Mali: analysis of prevalence and predictors. BMJ Open. 2023;13(4):e069226. 10.1136/bmjopen-2022-069226 PMC10151941 37094889

[52] Dela H, Attram N, Behene E, Kumordjie S, Addo KK, Nyarko EO, Letizia AG. Risk factors associated with gonorrhea and chlamydia transmission in selected health facilities in Ghana. BMC Infect Dis. 2019;19(1):425. 10.1186/s12879-019-4035-y PMC6524331 31096920

[53] Monroe-Wise A, Maman D, Edun O, Storey A, Lubega M, Carmone A, Johnson C. Successes and Setbacks in Dual HIV/Syphilis Test Scale-Up Strategies: Lessons Learned for Testing Integration in an Era of Declining Resources. 10.1016/j.hpopen.2026.100172 PMC13199886 42199455

[54] Khupe S, Salibi G, & Tzenios N. HIV prevalence in Southern African countries, complete with summaries and important points for each part . Spec J Med Acad Other Life Sciences. 2025;3(2). 10.58676/sjmas.v3i2.108Khupe.

[55] Jiang Z, Wang D, Yang S, Duan M, Bu P, Green A, et al. Integrated response toward HIV: a health promotion case study from China. Health Promot Int. 2011;26(2):196–211. 10.1093/heapro/daq044 20634201

[56] Sathiyasusuman A. Associated risk factors of STIs and multiple sexual relationships among youths in Malawi. PLoS ONE. 2015;10(8):e0134286. 10.1371/journal.pone.0134286 PMC4527764 26248328

[57] Chinsembu KC. Sexually transmitted infections in adolescents. Open Infect Dis J. 2009;3:107–17.

[58] Pathak A, Sawal A. Sexual Transmitted Disease among Youth. J Pharmaceut Res Int. 2021;30(60B). 10.9734/JPRI/2021/v33i60B34841.

[59] Dambach P, Mahenge B, Mashasi I, Muya A, Barnhart DA, Bärnighausen TW, Harling G. Socio-demographic characteristics and risk factors for HIV transmission in female bar workers in sub-Saharan Africa: a systematic literature review. BMC Public Health. 2020;20:1–14. 10.1186/s12889-020-08838-8 PMC7227324 32414352

[60] Maughan-Brown B, Evans M, George G. Sexual behaviour of men and women within age-disparate partnerships in South Africa: implications for young women’s HIV risk. PLoS ONE. 2016;11(8):e0159162. 10.1371/journal.pone.0159162 PMC4985138 27526116

[61] George G, Maughan-Brown B, Beckett S, Evans M, Cawood C, Khanyile D, Kharsany AB. Coital frequency and condom use in age-disparate partnerships involving women aged 15 to 24: evidence from a cross-sectional study in KwaZulu-Natal, South Africa. BMJ Open. 2019;9(3):e024362. 10.1136/bmjopen-2018-024362 PMC6429968 30852536

[62] Amsale Cherie AC, Yemane Berhane YB. Knowledge of sexually transmitted infections and barriers to seeking health services among high school adolescents in Addis Ababa, Ethiopia. 2012.

[63] Mahe A, Fonua L, Zablotska I, & Tin S. Knowledge, attitudes, and preventive practices towards sexually transmitted infections among youth in Tonga: local evidence to strengthen preventive actions. Med Res Arch. 2024;12(8). 10.18103/mra.v12i8.5600.

[64] Mensch BS, Grant MJ, Soler-Hampejsek E, Kelly CA, Chalasani S, Hewett PC. Does schooling protect sexual health? The association between three measures of education and STIs among adolescents in Malawi. Popul Stud. 2020;74(2):241–61. 10.1080/00324728.2019.1656282 PMC7162723 31619138

[65] Doyle AM, Changalucha J, Weiss HA, Watson-Jones D, Hayes RJ, Zaba B, et al. OP35 sexual risk behaviour among young people in rural Tanzania: do those in full-time education practise safer sex? J Epidemiol Community Health. 2013;67(Suppl 1):A18–9.

[66] Mee P, Fearon E, Hassan S, Hensen B, Acharya X, Rice BD, et al. The association between being currently in school and HIV prevalence among young women in nine eastern and southern African countries. PLoS ONE. 2018;13(6):e0198898. 10.1371/journal.pone.0198898 PMC6010266 29924827

[67] Badawi MM, SalahEldin MA, Idris AB, Hasabo EA, Osman ZH, Osman WM. Knowledge gaps of STIs in Africa; systematic review. PLoS ONE. 2019;14(9):e0213224. 10.1371/journal.pone.0213224. PMC6742237 31513584

[68] Mon Kyaw Soe N, Bird Y, Schwandt M, Moraros J. STI health disparities: a systematic review and meta-analysis of the effectiveness of preventive interventions in educational settings. Int J Environ Res Public Health. 2018;15(12):2819. 10.3390/ijerph15122819 PMC6313766 30544919

[69] Seidu AA, Aboagye RG, Okyere J, Adu C, Aboagye-Mensah R, Ahinkorah BO. Towards the prevention of sexually transmitted infections (STIs): healthcare-seeking behaviour of women with STIs or STI symptoms in sub-Saharan Africa. Sex Transm Infect. 2023;99(5):296–302. 10.1136/sextrans-2022-055424 PMC10359580 36202610

[70] Misinde C, Nansubuga E, Nankinga O. Out of school female adolescent employment status and sexually transmitted infections (STIs) risk in Uganda: is it a plausible relationship? BMC Public Health. 2018;18:1–10. 10.1186/s12889-018-6094-1 PMC6186056 30314473

[71] Lumbantoruan SM, Eka NGA, Saputri A. Self-reported sexually transmitted infections among women in Indonesia: analysis of 2017 Indonesia demographic and health survey. BMC Public Health. 2025;25(1):4088. 10.1186/s12889-025-25193-8 PMC12639969 41272629

[72] Aboagye RG, Seidu AA, Ahinkorah BO, Frimpong JB, Yaya S. Sexual violence and self-reported sexually transmitted infections among women in sub-Saharan Africa. J Biosoc Sci. 2023;55(2):292–305. 10.1017/S0021932022000062 35193714

[73] Aboagye RG, Ahinkorah BO, Seidu AA, Adu C, Hagan JE Jr, Amu H, et al. Mass media exposure and safer sex negotiation among women in sexual unions in Sub-Saharan Africa: analysis of demographic and health survey data. Behav Sci. 2021;11(5):63. 10.3390/bs11050063 PMC8145045 33925022

[74] Naidoo S, Wand H, Abbai NS, Ramjee G. High prevalence and incidence of sexually transmitted infections among women living in Kwazulu-Natal. South Africa AIDS research and therapy. 2014;11(1):31. 10.1186/1742-6405-11-31 PMC4168991 25243015

[75] Dong Y, Zhang H, Wang Y, Tao H, Xu S, Xia J, Operario D. Multiple abortions and sexually transmitted infections among young migrant women working in entertainment venues in China. Women Health. 2015;55(5):580–594. 10.1080/03630242.2015.1022811 PMC4559482 25902189

[76] De Jongh M, Lekalakala MR, Le Roux M, Hoosen AA. Risk of having a sexually transmitted infection in women presenting at a termination of pregnancy clinic in Pretoria, South Africa. J Obstet Gynaecol. 2010;30(5):480–3. 10.3109/01443611003797687 20604651

[77] Gokhale P, Madrigal JM, Aparicio J, Shim JY, Patel A. Demographic and other characteristics, and rates of sexually transmitted infections among adolescents who underwent multiple abortions in 1 year. J Pediatr Adolesc Gynecol. 2018;31(6):610–3. 10.1016/j.jpag.2018.07.011 30081083

[78] Osborne A, Essuman MA, Wongnaah FG, Aboagye RG, Bangura C, Ahinkorah BO. Provincial distribution and factors associated with self-reported sexually transmitted infections and their symptoms among women in Sierra Leone. BMC Infect Dis. 2024;24(1):1265. 10.1186/s12879-024-10030-x PMC11545995 39516716

[79] Amirkhanian YA. Review of HIV vulnerability and condom use in central and eastern Europe. Sex Health. 2011;9(1):34–43. 10.1071/SH11025 PMC4476380 22348631

[80] Ali Abdulai M, Baiden F, Afari-Asiedu S, Gyabaa-Febir L, Adjei KK, Mahama E, Owusu-Agyei S. The risk of sexually transmitted infection and its influence on condom use among pregnant women in the Kintampo north municipality of Ghana. J Sex Transm Dis. 2017;2017(1):8642685. 10.1155/2017/8642685 PMC5299183 28246570

[81] Crosby RA, Charnigo RA, Weathers C, Caliendo AM, Shrier LA. Condom effectiveness against non-viral sexually transmitted infections: a prospective study using electronic daily diaries. Sex Transm Infect. 2012;88(7):484–9. 10.1136/sextrans-2012-050618 PMC3502658 23002192

[82] Holmes KK, Levine R, Weaver M. Effectiveness of condoms in preventing sexually transmitted infections. Bull World Health Organ. 2004;82(6):454–61. PMC2622864 15356939

[83] Motsima T, Malela-Majika JC. The effects of early first sexual intercourse amongst Lesotho women: evidence from the 2009 Lesotho demographic and health survey. Afr J Reprod Health. 2016;20(2):34–42. 10.29063/ajrh2016/v20i2.4 29553162

[84] Ghebremichael M, Larsen U, Paintsil E. Association of age at first sex with HIV-1, HSV-2, and other sexual transmitted infections among women in northern Tanzania. Sex Transm Dis. 2009;36(9):570–6. 10.1097/OLQ.0b013e3181a866b8 PMC3529537 19707109

[85] Kaestle CE, Halpern CT, Miller WC, Ford CA. Young age at first sexual intercourse and sexually transmitted infections in adolescents and young adults. Am J Epidemiol. 2005;161(8):774–80. 10.1093/aje/kwi095 15800270

[86] Vasilenko SA, Kugler KC, Rice CE. Timing of first sexual intercourse and young adult health outcomes. J Adolesc Health. 2016;59(3):291–7. 10.1016/j.jadohealth.2016.04.019 PMC5002249 27265422

[87] Asekun-Olarinmoye OS, Asekun-Olarinmoye EO, Adebimpe WO, & Omisore AG. Effect of mass media and Internet on sexual behavior of undergraduates in Osogbo metropolis, Southwestern Nigeria. Adolesc Health Med Therapeut. 2014;5;15–23. https://doi-org.proxy.lib.uwaterloo.ca/10.2147/AHMT.S54339Asekun-Olarinmoye. 10.2147/AHMT.S54339 PMC3956478 24729737

[88] Coffee M, Lurie MN, Garnett GP. Modelling the impact of migration on the HIV epidemic in South Africa. AIDS. 2007;21(3):343–50. 10.1097/QAD.0b013e328011dac9 17255741

[89] Rahman M, Nakamura K, Seino K, Kizuki M. Intimate partner violence and symptoms of sexually transmitted infections: are the women from low socio-economic strata in Bangladesh at increased risk. Int J Behav Med. 2014;21(2):348–57. 10.1007/s12529-013-9302-5 23515966

[90] Harling G, Subramanian SV, Bärnighausen T, Kawachi I. Socioeconomic disparities in sexually transmitted infections among young adults in the United States: examining the interaction between income and race/ethnicity. Sex Transm Dis. 2013;40(7):575–81. 10.1097/OLQ.0b013e31829529cf PMC3752095 23965773

